# Quantitative Assessment of the Effects of Compression on Deep Learning in Digital Pathology Image Analysis

**DOI:** 10.1200/CCI.19.00068

**Published:** 2020-03-10

**Authors:** Yijiang Chen, Andrew Janowczyk, Anant Madabhushi

**Affiliations:** ^1^ Case Western Reserve University, Cleveland, OH; ^2^ Precision Oncology Center, Lausanne University Hospital, Lausanne, Switzerland; ^3^ Louis Stokes Cleveland Veterans Affair Medical Center, Cleveland, OH

## Abstract

**PURPOSE:**

Deep learning (DL), a class of approaches involving self-learned discriminative features, is increasingly being applied to digital pathology (DP) images for tasks such as disease identification and segmentation of tissue primitives (eg, nuclei, glands, lymphocytes). One application of DP is in telepathology, which involves digitally transmitting DP slides over the Internet for secondary diagnosis by an expert at a remote location. Unfortunately, the places benefiting most from telepathology often have poor Internet quality, resulting in prohibitive transmission times of DP images. Image compression may help, but the degree to which image compression affects performance of DL algorithms has been largely unexplored.

**METHODS:**

We investigated the effects of image compression on the performance of DL strategies in the context of 3 representative use cases involving segmentation of nuclei (n = 137), segmentation of lymph node metastasis (n = 380), and lymphocyte detection (n = 100). For each use case, test images at various levels of compression (JPEG compression quality score ranging from 1-100 and JPEG2000 compression peak signal-to-noise ratio ranging from 18-100 dB) were evaluated by a DL classifier. Performance metrics including F1 score and area under the receiver operating characteristic curve were computed at the various compression levels.

**RESULTS:**

Our results suggest that DP images can be compressed by 85% while still maintaining the performance of the DL algorithms at 95% of what is achievable without any compression. Interestingly, the maximum compression level sustainable by DL algorithms is similar to where pathologists also reported difficulties in providing accurate interpretations.

**CONCLUSION:**

Our findings seem to suggest that in low-resource settings, DP images can be significantly compressed before transmission for DL-based telepathology applications.

## INTRODUCTION

The advent of whole-slide scanners has enabled high-throughput digitization of routine glass pathology tissue slides. The digitization of glass slides, or digital pathology (DP), has in turn also enabled digital transmission of DP slides over the Internet for secondary diagnosis, a practice termed telepathology (TP).^1,2^ TP has been implemented in a variety of applications including primary histopathology diagnoses,^3^ second opinions, subspecialty consultations, and intraoperative frozen section services.^1^ TP systems require hardware for slide digitization (eg, slide scanner or microscope camera) linked to a computer with Internet access,^4^ which enables a pathologist at a remote location to then view and interpret the digitized slide image.

DP slides can also be analyzed by deep learning (DL), a machine learning approach that recognizes patterns in DP images through a network of connected artificial neurons. One of the most popular DL network types is the convolutional neural network (CNN).^5,6^ Through an iterative examination of a labeled data set, CNNs attempt to learn increasingly higher levels of data abstractions from the original data. This process, which involves minimizing the error between the model prediction and ground truth data labels, allows for learning the most discriminating representations between categories of interest. CNNs have been proposed to increase the efficiency of tasks such as segmentation of histologic primitives (eg, nuclei segmentation^5^ and epithelium segmentation^7^), detection (eg, mitotic events^8^), disease identification/localization (eg, cancerous *v* noncancerous),^1^ and disease diagnosis.^2^ Recently, DL approaches have been used to identify tissue primitives such as nuclei and tubules from which morphologic features (eg, shape, texture, arrangement) can be extracted and further associated with disease prognosis, outcome, and treatment response.^9,10^

Although there has recently been a great deal of interest in developing and applying DL approaches in DP, the question of the effect of image compression on DL algorithms has been largely unexplored.^11^ Compression technologies are especially important in countries with poor quality Internet access, where sending and receiving large DP image files can be challenging.^12,13^ For instance, a single prostate biopsy slide digitized at 40× can easily result in > 5 gigabytes of data, with a typical pathology workflow requiring approximately 12 slides. To help alleviate the storage and transmission burdens in TP, image compression seems to be the logical solution to reduce the size of DP files.

Previous work has focused on assessing the effects of common lossy image compression algorithms on DL performance.^14,15^ On the basis of a similar experimental methodology, we sought to evaluate how different degrees of image compression affect CNNs in the use cases of nuclei (n = 137) and lymph node metastasis segmentation (n = 380) and lymphocyte detection (n = 100). Our approach involved training DL networks using high-fidelity images and subsequently evaluating model performance using held-out test sets subjected to increasing levels of either JPEG or JPEG2000 compression. Additionally, attempts were made to identify the maximum compression level beyond which the CNN, and pathologist, interpretations began to substantially degrade. We also sought to evaluate the changes in nuclei-derived image features (eg, cell distribution graph) as a function of DL performance over different compression levels. CNNs were chosen for this study because they currently represent the most popular DL approach in the DP space. The selected use cases were chosen because of their similarity to commonly performed DP tasks.^5^

## METHODS

### Experimental Pipeline

**FIG 1.**
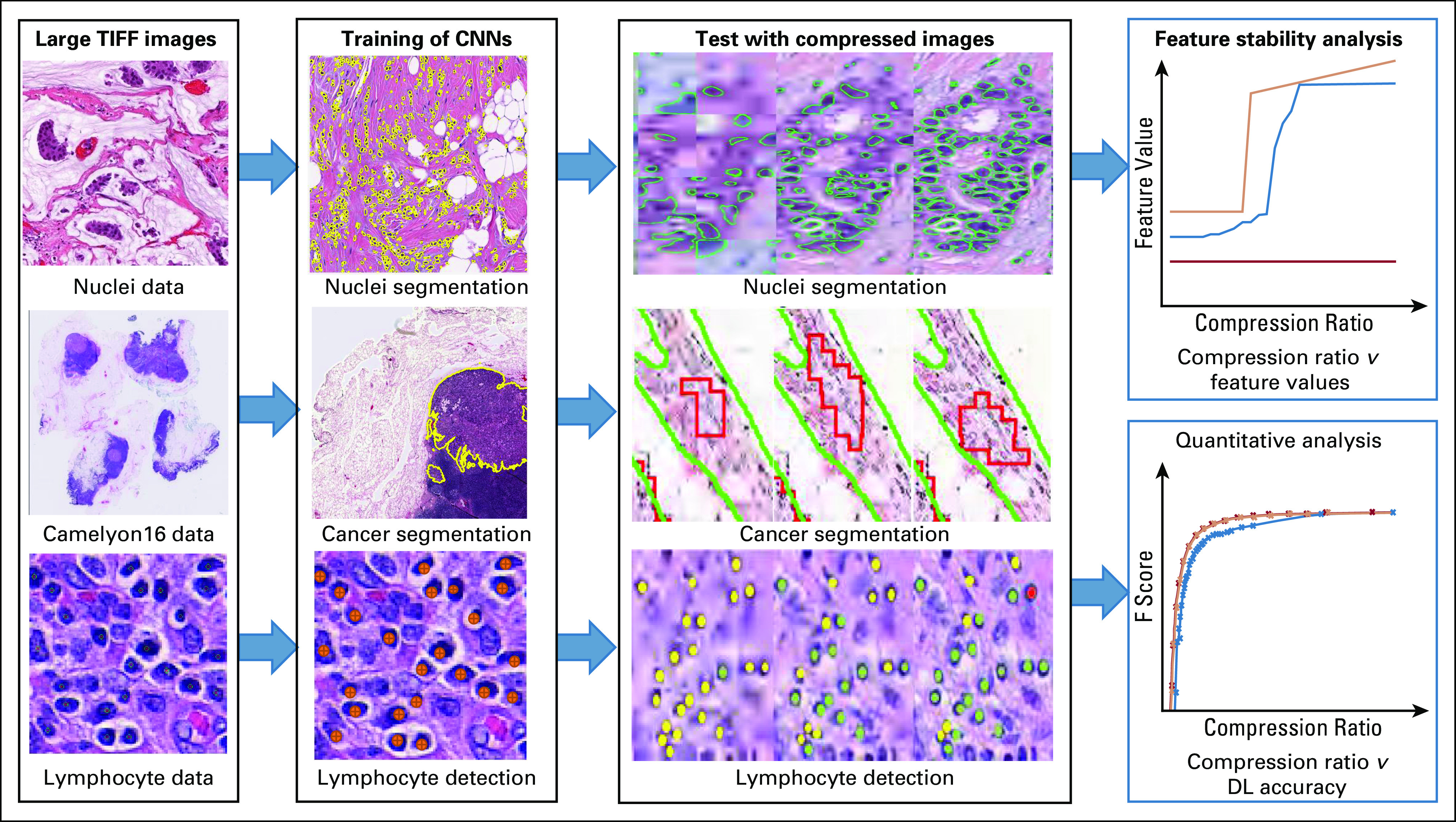
Flowchart illustrating the experimental design for segmentation of (1) nuclei (n = 137) and (2) lymph node metastasis (n = 380), and (3) detection of lymphocytes (n=100). For each use case, images were divided into training and testing sets. High-quality tissue images cropped from whole-slide images were used for deep learning (DL) classifier training. Varying levels of lossy compression were applied to test images for evaluation. For each use case, deep learning performance on the compressed test images across various compression ratios was quantitatively evaluated. For the nuclei segmentation use case, primitive derived image features (eg, features relating to spatial arrangement of nuclei) were extracted and assessed for stability across compression levels. CNN, convolutional neural network.

In this work, we sought to quantitatively evaluate the effect of different degrees of compression on DL classifiers via 3 use cases: nuclei segmentation, lymph node metastasis segmentation, and lymphocyte detection. Each use case followed the pipeline illustrated in Figure 1. Briefly, for each use case, an AlexNet^16^ (ie, a type of CNN) was trained using high-fidelity regions of interest (ROIs) cropped from whole-slide images (WSIs) generated by Aperio scanners (Leica Biosystems, Nussloch, Germany) using default settings. During training, data set augmentation was enacted by random rotations of {0, 90, 180, 270}, along with random mirroring (details regarding training of each classifier can be found in the Appendix). Subsequently, held-out test images were subjected to increasing levels of compression, and the relationship between compression level and a number of quantitative performance metrics (eg, pixel-level F1 score, object detection F1 score, and pixel-level area under the receiver operating characteristic curve [AUC]) was studied.

#### Quantitative metrics for evaluating DL performance.

The nuclei segmentation and lymphocyte detection use cases used the F1 score^5^ (or F score), where 0 indicates worst performance and 1 indicates best performance for the classifier. For lymph node metastasis segmentation, pixel-level AUC^1^ was used. Image degradation was measured using the peak signal-to-noise ratio (PSNR).^17^ This study examined ranges of PSNR from 18 to 100 dB, with 18 dB being the lowest computable by the OpenJPEG library (version 2.3.1)^18^ resulting in maximal compression and 100 being lossless. Intuitively, strong negative correlation exists between PSNR and the compressed image size.^19^

#### Evaluation of feature stability from nuclear segmentations.

Graph, nuclear, and subgraph features were derived from the nuclear segmentation output. A total of 77 first-order summary statistics were subsequently computed and their stability in the presence of compression evaluated. The graph features aim to model global cellular spatial distribution via various algorithms (eg, Voronoi diagram, Delaunay triangulation, minimum spanning tree). Nuclear features pertaining to spatial distribution as well as morphologic appearance (eg, size, eccentricity, nearest neighbor properties) were also calculated. Finally, subgraph features reflecting local cellular distribution of cells via clustering algorithms (eg, connected components) were also calculated. These features have been shown to hold diagnostic and prognostic value in the context of various diseases.

#### Pathologist evaluation of compressed images.

We also sought to evaluate the degree of image compression that a pathologist could tolerate in performing the same 3 use cases: segmentation of nuclei and lymph node metastasis and detection of lymphocytes. Toward this end, 3 pathologists were asked to examine 10 randomly chosen test images per use case at each level of compression. They subsequently reported the highest compressed level for which they would feel comfortable performing the assigned segmentation/detection task (ie, tracing nuclear boundaries, identifying lymphocytes, or delineating cancerous regions). Each pathologist involved in this study was tasked with the review of a single use case.

### Image Compression Approaches

JPEG and JPEG2000^20^ lossy compression approaches were used in this study because they are most commonly used by scanner manufacturers and WSI formats.^19^ JPEG allows the user to specify the desired level of compression via a quality score associated with the quantization of frequencies in the image. When employing JPEG2000, the user specifies a PSNR value, which results in the truncation of certain frequencies after a wavelet transformation. These lossy compression algorithms achieve their reduction performance by eliminating high-frequency image features (eg, noise, subtle textures), which tends to result in blurring and distortion at higher compression levels. In all cases, JPEG2000 demonstrated superior performance and additionally allowed for a lossless 40% to 70% reduction in file size depending on the image content. Details regarding JPEG/JPEG2000 can be found in the Appendix.

## RESULTS

### Use Case 1: Nuclei Detection and Segmentation

#### Data set description.

WSIs of 137 patients with estrogen receptor (ER) –positive breast cancer were scanned at 40× using an Aperio whole-slide scanner and saved using the scanner default quality score of 70%. From this cohort, 143 2,000 × 2,000 ROIs containing cancer were extracted. From these regions, approximately 12,000 nuclei were manually annotated and confirmed by a pathologist. Patients were randomly assigned to training and testing groups at a ratio of 8:2.

#### Compression experiment results.

##### DL results.

**FIG 2.**
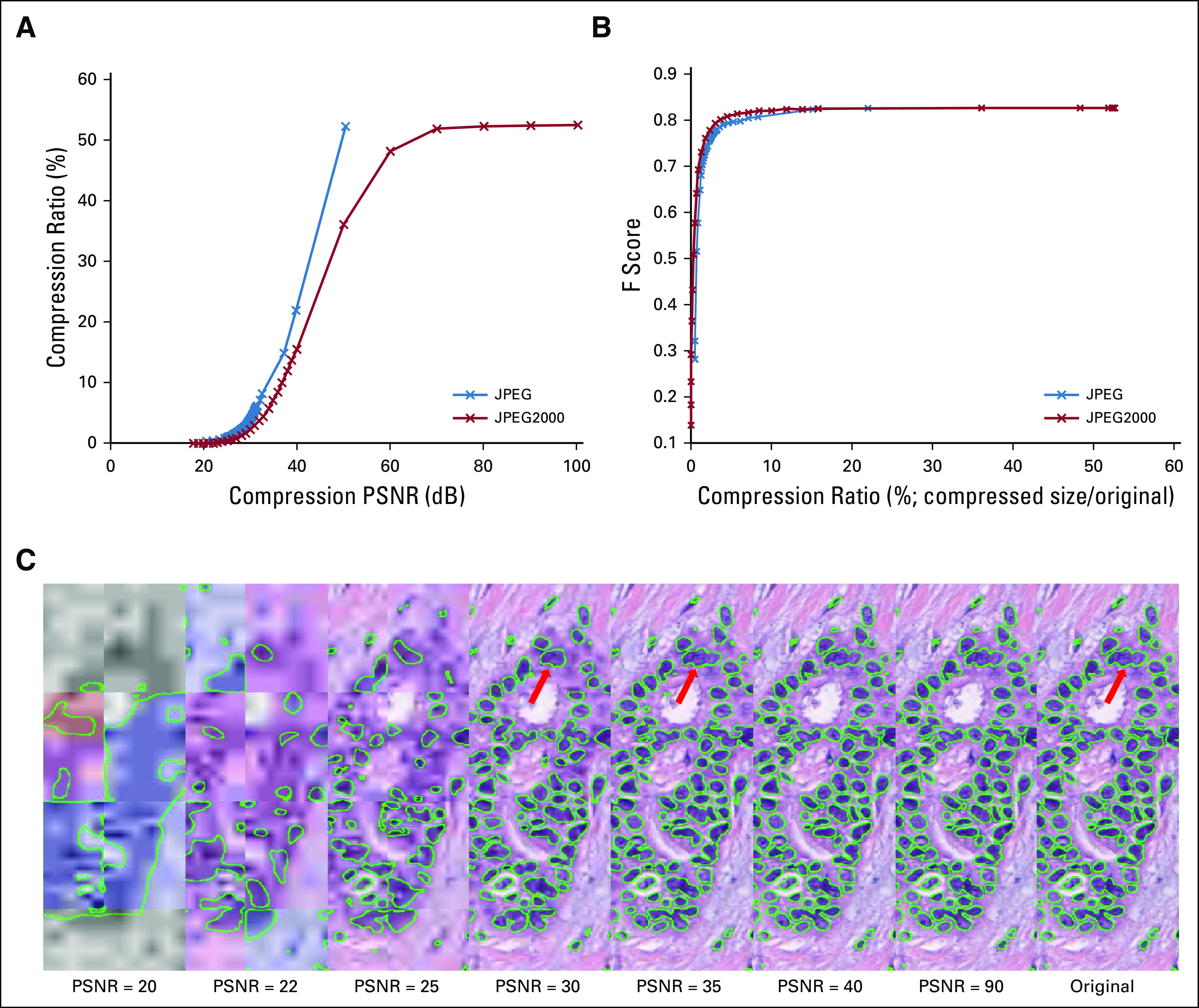
(A) Graph showing the strong negative relation between peak signal-to-noise ratio (PSNR) and compression ratio (compressed size divided by original file size) for different types of compression (JPEG and JPEG2000). JPEG2000 appears to show superiority over the JPEG compression format in terms of compression efficiency. (B) Graph showing degradation of F-score for test images against compression ratio for different types of compression. (C) An image subjected to varying levels of compression (PSNR = 20, 22, 25, 30, 35, 40, 90, and lossless) with the resulting DL output overlaid in green. The nuclear segmentation results appear to notably degrade when the PSNR is < 30 (highlighted in red arrow).

The DL model was trained at 10× magnification and produced a pixel-level F score of 0.83 on the uncompressed held-out test images. Although increasing compression (PSNR from 100 to 18 dB) resulted in decreases in segmentation performance, this decrease was notably small until the compression ratio fell below 5% (Figs 2A and 2B). Below this level, the segmentation performance dropped dramatically (an exponential drop from 0.83 to 0.3 in F score). As the compression ratio progressed from 53% (lossless compression) to 3%, the overall average segmentation results only deteriorated by 3.5% (ie, F score decreased from 0.83 to 0.80; Fig 2B).

##### Pathologist evaluation of compressed images.

Interestingly, the pathologists identified a PSNR of 30 dB as the maximum compression level they would feel comfortable performing the same segmentation task. This corresponds to a compression ratio of 3% and is close to the point at which performance of the DL classifier starts to degrade dramatically (Fig 2B). Higher compression levels resulted in ambiguous nuclear boundaries and thus would have prevented accurate annotation.

##### Evaluating variability in extracted nuclear features as function of compression levels.

A set of well-documented features used in DP image analysis^21^ were next employed. These included cellular graph features (eg, Voronoi diagram, Delaunay triangulation, minimum spanning tree, cell cluster subgraph), which focus on capturing global and local spatial cellular architecture, as well as nuclear features, which focus on nuclear morphology (eg, shape and texture). Our hypothesis stated that increasing levels of compression may cause the DL model to potentially miss or incorrectly identify the boundaries of nuclei, thus imparting variability during feature computation.

**FIG 3.**
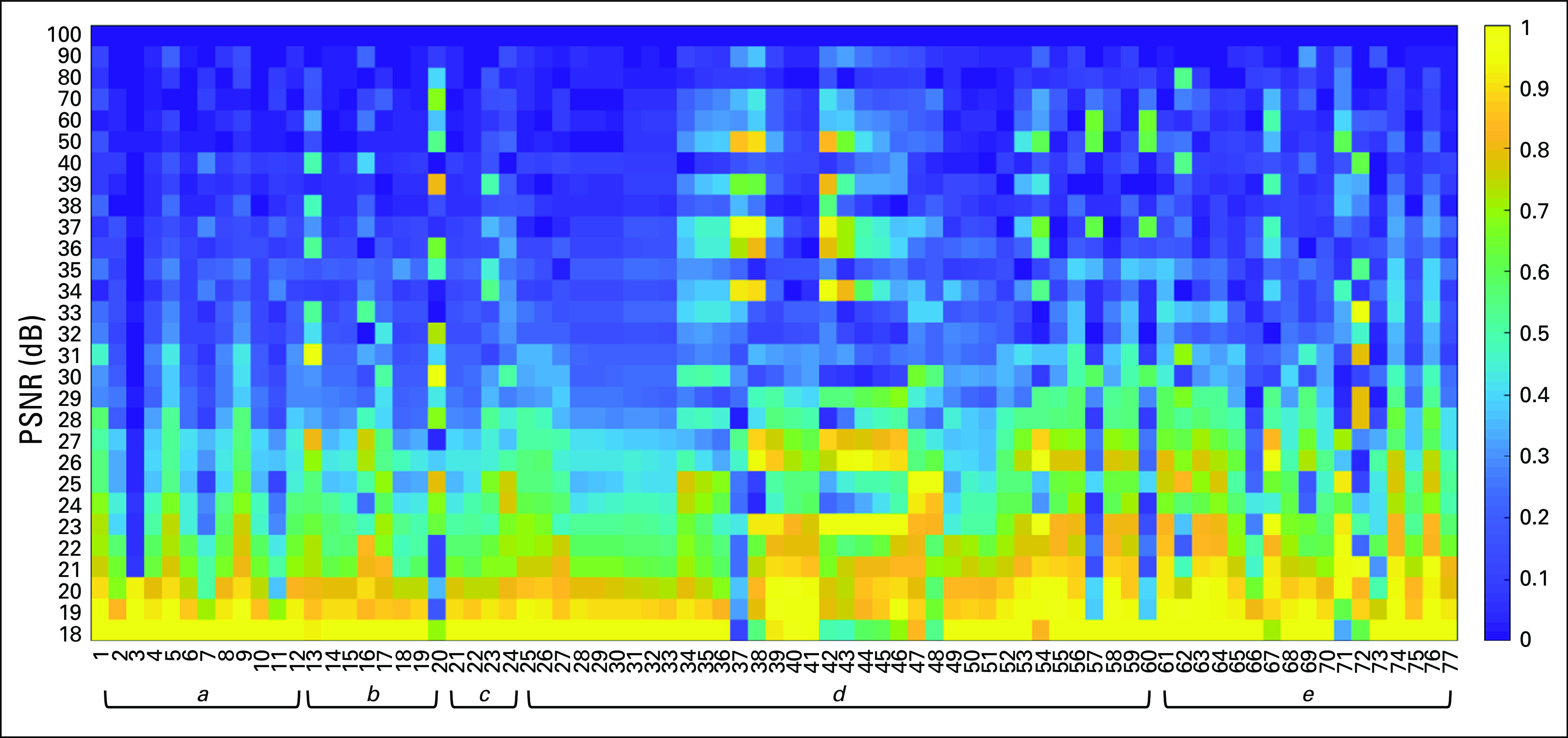
Heat map, grouped by feature families, showing the relative difference (0,1) of each feature (x-axis, feature names in the Appendix) at different levels (peak signal-to-noise ratio = 18 dB-100 dB) of compression (y-axis) as compared to values derived from the original images. The higher the relative difference, the more the feature appears to be sensitive to image compression. It was observed that some first-order statistical features (eg, standard deviation, min, max) do not deviate in a monotonic fashion as a function of image compression. a, Voronoi feature; b, Delaunay triangulation; c, minimum spanning tree; d, nuclear feature; e, cell cluster subgraph feature.

Figure 3 illustrates that the global graph feature^22,23^ family, which contains features such as Voronoi diagram, minimum spanning tree, and nuclear distribution, stayed relatively stable when the PSNR is > 40 dB. Conversely, most of the subgraph features seemed unstable in the presence of compression artifacts. A reference table of each feature can be found in the Appendix.

### Use Case 2: Breast Lymph Node Metastasis Segmentation

#### Data set description.

To aid in appreciating how compression may affect popular challenge data sets, the publicly available Camelyon16^24^ data set was used. This data set comprises 400 WSIs at 40× magnification, in TIFF format, divided into 270 images for training/validation and 130 for testing.

#### Compression experiment results.

##### DL results.

**FIG 4.**
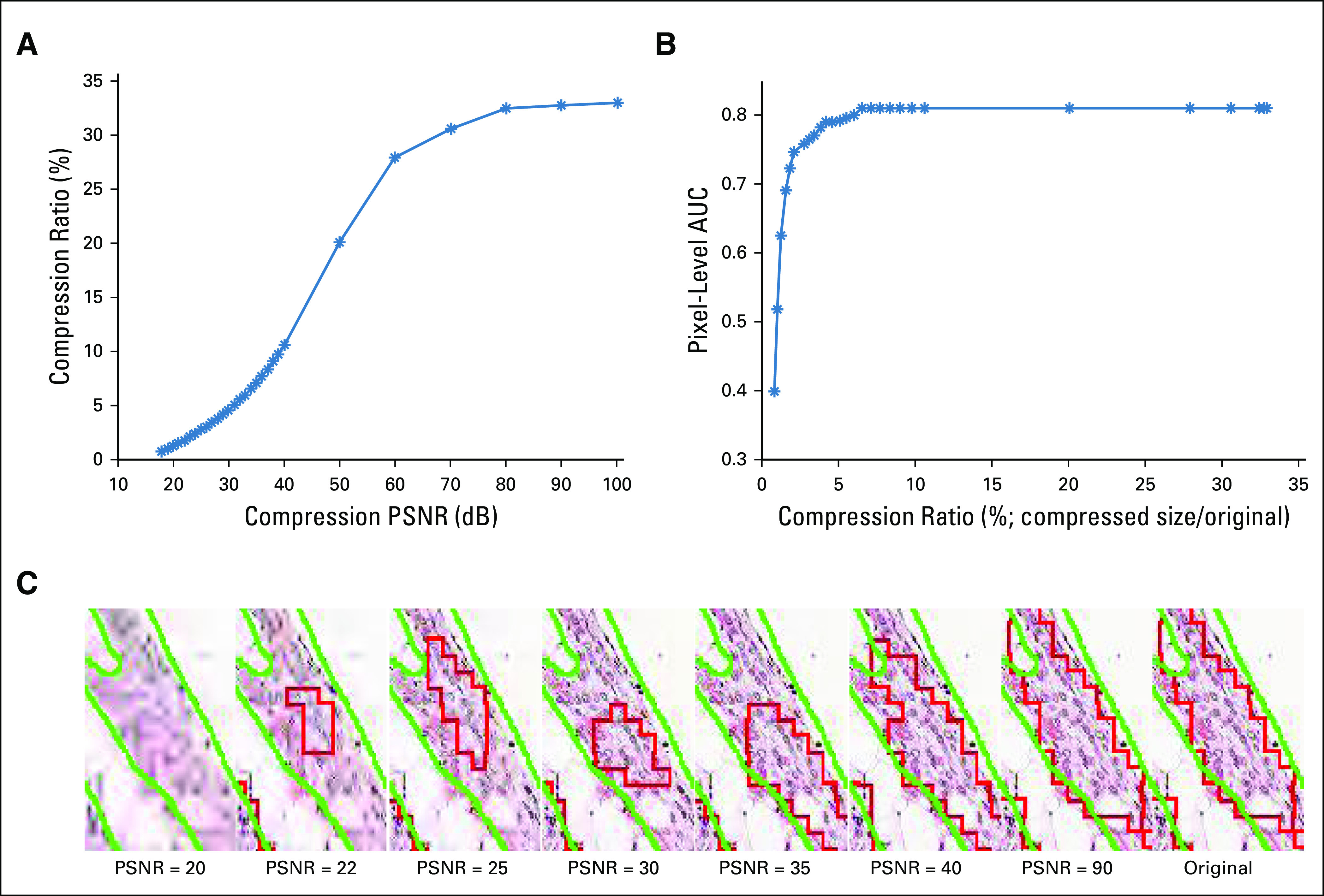
(A) Graph showing the strong negative relation between peak signal-to-noise ratio (PSNR; 18 dB-100 dB) and compression ratio (compressed size divided by original file size) for JPEG2000 compression. (B) Graph showing degradation of pixel-based area under the curve for test images with various compression ratios for JPEG2000 compression. (C) A region of interest illustrating effects from varying levels of compression (PSNR = 20, 22, 25, 30, 35, 40, 90, and lossless). The deep learning output overlaid in red, with ground truth overlaid in green, shows the boundary of the detected tumor region changing drastically as the PSNR drops below 35 dB.

**FIG 5.**
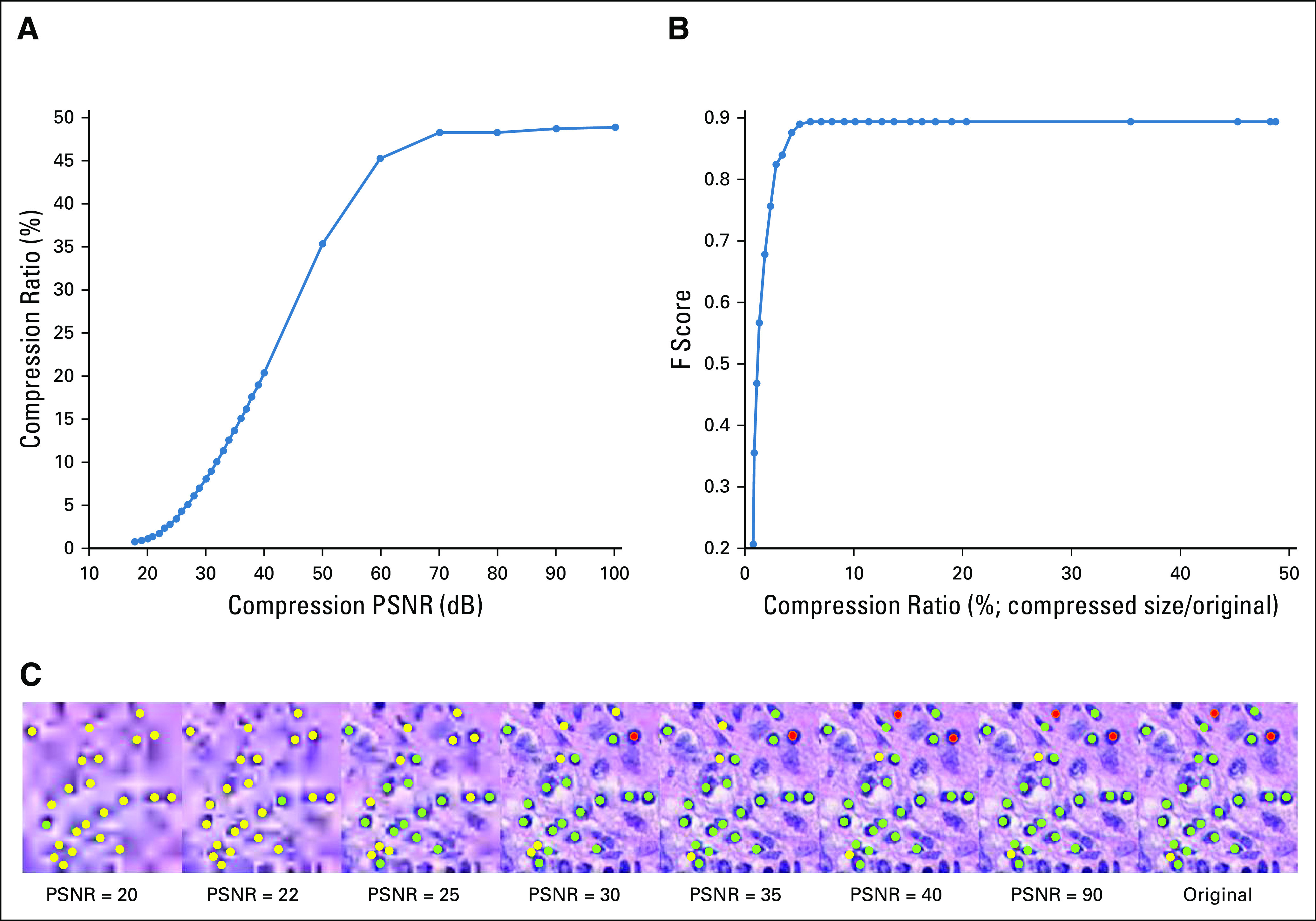
(A) Graph showing the strong negative relation between peak signal-to-noise ratio (PSNR; 18 dB-100 dB) and compression ratio (compressed size divided by original file size in percentage) for JPEG2000 compression. (B) Graph showing degradation of cell-based F-score for test images with various JPEG2000 compression ratios. (C) An image subjected to varying levels of compression (PSNR = 20, 22, 25, 30, 35, 40, 90, and lossless) with the resulting deep learning output from the lymphocyte detection model overlaid. A green label indicates a correctly detected lymphocyte, a red label indicates a false positive, and a yellow indicates a false negative. As observed in this figure, the morphology of lymphocytes started to change drastically after the PSNR decreases to < 30 dB, coinciding with an 8% compression ratio.

The DL model was trained at 5× magnification and demonstrated a pixel-level AUC of 0.92 in the validation cohort and 0.81 in the test cohort. These results are comparable to other state-of-the-art breast lymph node metastasis segmentation approaches.^25,26^ Although increasing compression (PSNR from 100 to 18 dB) resulted in decreases in segmentation performance, this decrease was notably small until the compression ratio fell below 4% (Figs 4A and 5B). Below this level, the segmentation performance exponentially dropped from 0.8 to 0.4 in pixel-level AUC. As the compression ratio progressed from 33% (lossless compression) to 4%, the overall average segmentation results only deteriorated by 2% (ie, AUC decreased from 0.81 to 0.79; Fig 5).

The relationship between compression ratio and PSNR was similar to that of the other use cases (Figs 4A and 4B). In contrast, a maximum total difference of 0.4 AUC was demonstrated between the highest and lowest levels of compression, with a slower drop in model performance at the intermediate compression levels (2%-35% compression ratio) compared with both the nuclear and lymphocyte use cases, suggesting this use case was the least affected by higher compression levels.

##### Pathologist evaluation of compressed images.

The pathologists reported a PSNR of 22 dB (corresponds to a compression ratio of 1%) as the maximal compression level that would allow them to confidently segment the regions of lymph node metastasis. Similar to the nuclear segmentation use case, any PSNR lower than this value induced too much uncertainty in identifying the cancer boundary. This PSNR level is lower than that for acceptable DL performance (pixel-level AUC of 0.79 at 4% compression ratio) but still within a comparable range.

### Use Case 3: Lymphocyte Detection

#### Data set description.

The lymphocyte detection data set consisted of 100 ER-positive breast cancer images (100 × 100) cropped from WSIs scanned at 20× and saved using the scanner default quality score of 70%. The centers of 3,064 lymphocytes were identified and labeled on the images by a pathologist.^5^ The data set was divided into training and testing sets at a ratio of 8:2.

#### Compression experiment results.

##### DL results.

The DL model was trained at 20× magnification and produced a cellular-level detection F score of 0.896 on the uncompressed held-out test images. Similar behavior with other use cases between compression ratio and both PSNR and F score was observed (Figs 5A and 5B). The decrease in F score was notably small until the compression ratio fell below 5%. Below this level, the detection performance experienced an exponential drop from 0.89 to 0.2 in F score. As the compression ratio progressed from 49% (lossless compression) to 5%, the overall average detection results only deteriorated by < 0.5%, indicating the high tolerance of the model to JPEG2000 compression artifacts. The model maintained an F score > 0.8 even when images were compressed to 3% of their original size.

##### Pathologist evaluation of compressed images.

The pathologists reported a PSNR of 30 dB (corresponds to a compression ratio of 7%) as the maximum compression level that would allow them to confidently differentiate lymphocytes from other types of cells. Any PSNR lower than this value could cause false identification, likely as a result of color and edge distortions. This PSNR level is higher than that for acceptable DL performance (detection F score of 0.89 at 5% compression ratio) but still within a comparable range.

## DISCUSSION

Uncompressed, a typical WSI of 200,000 × 200,000 would require > 120 gigabytes of storage. Furthermore, a single intervention may result in multiple slides being generated (eg, prostate biopsy procedures routinely result in up to 12 different tissue slides being prepared and interrogated). Taken together, it is likely that these images will need to undergo significant compression for both transmission and storage. This will be especially critical in the context of TP applications in countries with limited Internet infrastructure and bandwidth.^1-3,27,28^ These facilities also tend to lack sufficient computational resources to autonomously develop and deploy DL approaches. With a sufficient reduction in transmission overhead, however, it may be possible to more routinely use TP as a service. This would see expert centers developing computational approaches and providing associated infrastructure so that others may leverage those models via the uploading of their WSIs. To minimize the storage and transfer burdens associated with these DP images, one would ideally like to identify the maximal level of compression possible while not sacrificing diagnostic performance, both from the context of human diagnostic and machine learning perspectives.

DL is becoming increasingly popular in the context of DP.^5^ Although recent research has investigated the effects of compression on DL performance in natural images,^15^ relatively little study has taken place in dp-based image analysis tasks.^11,29^ We aimed to address that need by studying the inverse relationship between compression and performance of DL algorithms in DP images.

This work examined the effects of compression on DL in 3 representative use cases: nuclei segmentation (n = 137), lymph node metastasis segmentation (n = 380), and lymphocyte detection (n = 100). In all evaluated use cases, our results suggest that JPEG2000 is superior to JPEG for DP images. With JPEG2000 compression, file size could be reduced by > 80% with almost no loss in DL or pathologist performance irrespective of use case. Furthermore, files could be compressed by 95% with < 2% loss in segmentation and detection performance. In particular, images containing large homogeneous areas (ie, background) exhibited higher compression ratios with less performance loss. Features extracted from nuclei became significantly compromised when the PSNR dropped below 40 dB, likely because of changes in tissue texture and ambiguity in nuclear morphology imparted by higher levels of compression. Global graph features showed the most resilience to compression as a result of their overall robustness to incorrect nuclei detection. In general, the features that were least stable to compression tended to model small areas of tissue. Similarly, less dramatic performance degradation was observed during the metastasis segmentation use case, potentially as a result of the lower 5× magnification being used. At this magnification, more resilient higher-level image features (eg, entropy, texture, color) are more prevalent rather than detailed properties derived from cellular morphology, which tend to degrade quickly at higher levels of compression.

In general, there is a careful balance to be found between the quality and size of DP images when employing compression. Our experiments show a PSNR of 40 dB (approximately 1:10 compression ratio based on the image) results in almost no loss in DL performance. With task-specific validation, a PSNR of 35 dB (approximately 1:20 compression ratio based on the image) may still allow robust DL performance with notably smaller file sizes. Interestingly, the pathologists’ minimum-needed compression level for a confident read was concordant with the level beyond which DL model performance deteriorated significantly. Last, augmenting the training set with a range of compressed images did not seem to improve DL performance on images compressed within the range of 30 to 40 db (F score of +.01), moderately improved DL performance on more heavily compressed images (F score of +0.05 to 0.093 in PSNR range 21-27 dB), and resulted in minor improvement on intensely compressed images (F score of +0.005 to 0.039 in PSNR range 18-20 dB; Appendix).

A recent study by Zanjani et al^11^ evaluated the impact of JPEG2000 compression on a DL model for slide-based breast lymph node metastasis detection. Our study confirms their findings: their DL models seemed to be robust up to a compression ratio of 1:24 (96% reduction in size). Another related study by Doyle et al^29^ evaluated both the performance of the pathologist and that of a machine classifier to detect prostate cancer on JPEG2000 compressed DP images, although the machine classifier was not a DL approach. Our study confirmed the conclusion from their reader inspection experiments: the compression threshold reported by their pathologist was in line with the threshold reported by our pathologists (99.2% reduction in size through JPEG2000 compression in metastatic cancer segmentation in our study *v* 98.5% reduction in prostate cancer diagnosis reported by Doyle et al^29^).

Our approach in this study differed from that of Zanjani et al^11^ and Doyle et al^29^ in the following 2 ways. Firstly, our study and that by Zanjani et al^11^ assessed the impact of lossy compression on DL, whereas Doyle et al^29^ explored the impact of JPEG2000 compression on a handcrafted machine learning approach. Compared with the work of Doyle et al^29^, the DL models were shown to be less robust to compression artifacts than their CAD system. In both our study and the Zanjani et al^11^ study, the maximum compression allowed for confident performance (< 3% loss in accuracy) from DL models was marginally smaller than that reported by Doyle et al^29^ (99.6% *v* < 97% reduction in size). An explanation for this performance discrepancy may be that the machine classifier used by Doyle et al^29^ performed cancer classification based solely on larger histologic primitives, such as the size and location of gland lumen. These high-level features tend to be robust under heavy JPEG2000 compression, even though minute tissue details (eg, textural features) are severely compromised. Secondly, our study had a wider scope, because it explored 3 of the most common distinct DL-based use cases in DP, covering both segmentation as well as detection tasks. Real-world implementations of compression will need to address the unique properties of each task.

Experiments were conducted using a NVidia Titan X GPU (Santa Clara, CA). For use cases 1 and 3, model training required approximately 3 hours, with output generation per test image taking approximately 1 second. For use case 2, because of the larger data set, 6 hours were required to train the model, with approximately 1 minute needed to generate output for each patient.

Our study did have limitations. Firstly, it is evident that DL performance in the presence of compression artifacts is task specific. Although the 3 use cases studied here are representative of many DP tasks, they are by no means exhaustive, because there are many other DP applications of DL, including tissue classification, outcome prediction, and treatment response prediction. On the basis of the results of this study, we recommend that in all cases, a compression level resulting in a PSNR < 40 dB be evaluated carefully. Secondly, only JPEG and JPEG2000 compression algorithms were considered for evaluation in our study. To our knowledge, most WSI slide-scanner manufactures use 1 of these 2 approaches as the backbone of their proprietary formats.^19,30,31^ As a result, their study is most likely to be relevant in the storage and transmission of WSI images. That said, as more powerful novel compression schemes are introduced,^32^ tested, and routinely used in practice, future work will be required to evaluate their respective compression artifacts. Last, in our pathologist evaluation study, each pathologist was tasked with visual assessment of 1 single use case. This is potentially a limitation of our study. However, it seems in experimental results that compression artifacts are subtly added to an image as compression levels are increased until they reach a breaking point, after which the changes are abruptly severe. This abrupt change in image quality may potentially explain the low interexperiment variability observed in both pathologists and DL models, suggesting low subjectivity in human reader judgments.

In spite of these limitations, our study is the first comprehensive attempt to our knowledge to quantitatively evaluate the effects of image compression on DL algorithms across a variety of different use cases in the DP domain. It is our hope that the findings in this study can serve as a guide to identifying the appropriate degree of image compression for both DP image analysis and TP-specific tasks.

## Figures and Tables

**FIG A1.**
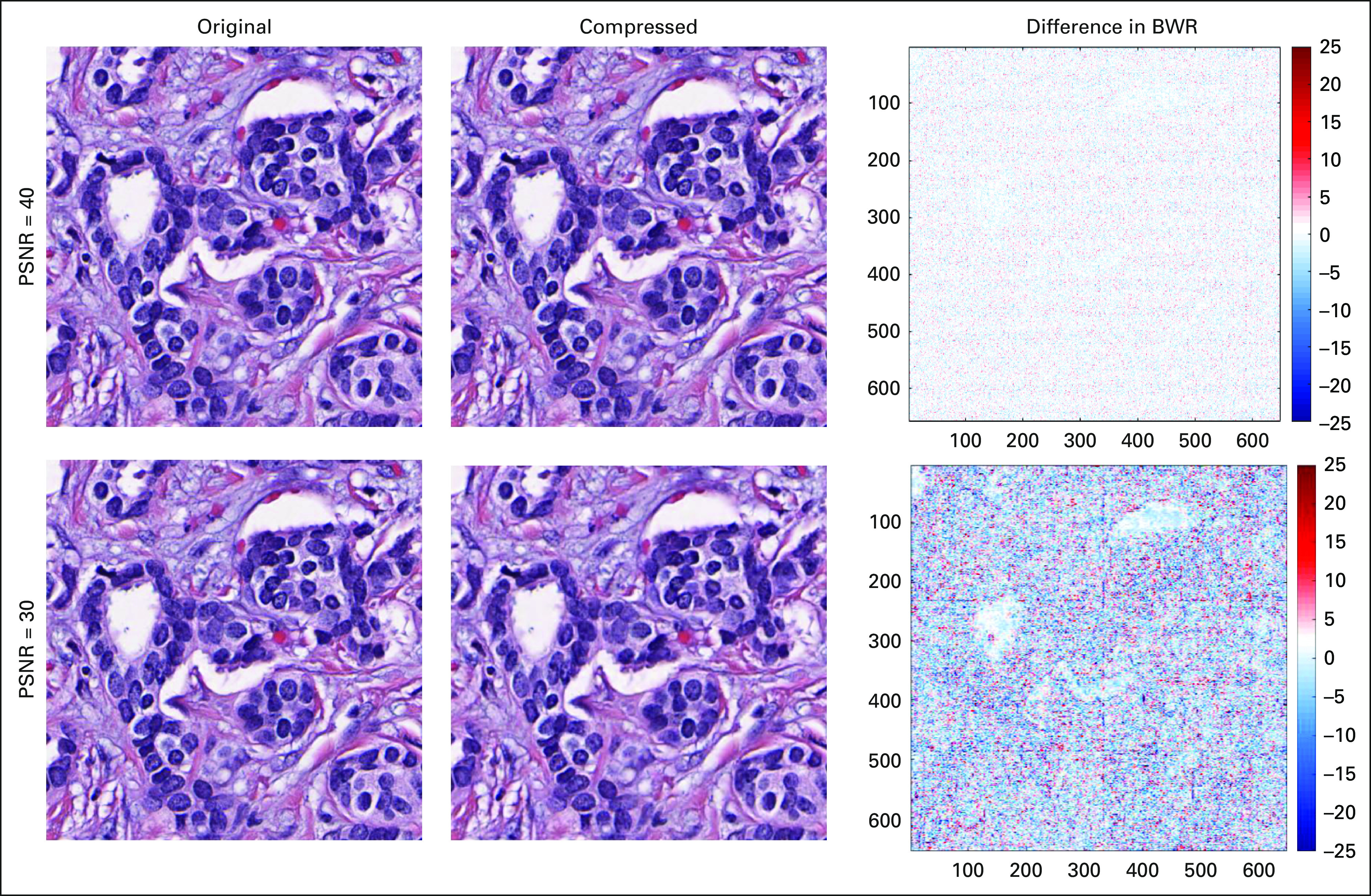
An illustration of the compression artifacts introduced into the original image at (top row) peak signal-to-noise ratio (PSNR) of 30 dB and (bottom row) PSNR of 40 dB. The left column shows the uncompressed input image, the middle column shows the associated compressed image, and the final column shows the difference between the original and compressed images after conversion to grayscale. The difference map is shown using a divergent isoluminant blue-white-red (BWR) color map for easier visualization. As can be observed, difference between PSNR of 40 and 30 dB is even greater than the difference between PSNR of 40 dB and the original image. When compared with the original image, the image of PSNR of 40 dB showed only little compression noise of 0 to 4 difference in pixel value of 255 maximum in unit8. However, when the image is compressed to a PSNR of 30 dB, much more intense compression artifacts can be observed on nonwhite regions.

**FIG A2.**
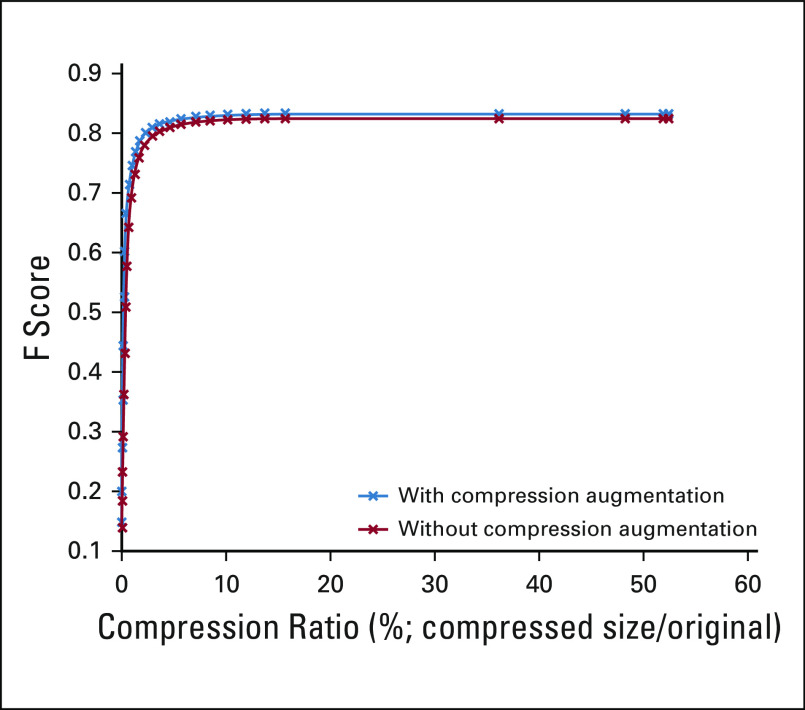
Segmentation F score of nuclei segmentation (use case 1) models trained with and without using JPEG2000 compression as a form of augmentation plotted on the same coordinate. As can be observed, a minor improvement in F score was achieved for lightly compressed images, whereas a more significant improvement in F score was observed for more heavily compressed images.

**FIG A3.**
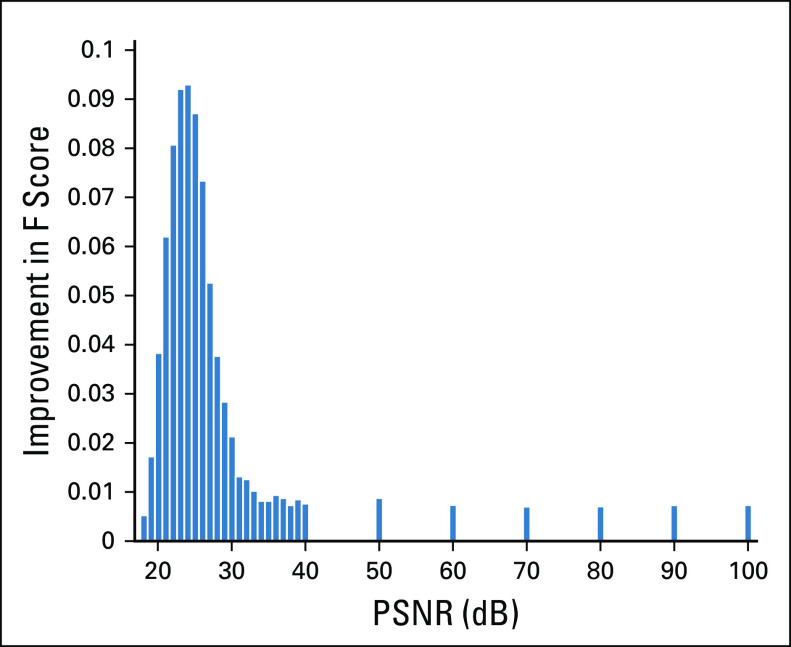
The improvement of F score using lossy compression as a form of augmentation plot for different compression peak signal-to-noise ratios (PSNRs) explored. As observed, lossy compression augmentation improved the segmentation performance marginally (< 0.015) on lightly compressed images with PSNR between 30 and 100 dB, whereas an improvement of 0.005 to 0.093 in normal distribution was observed for images compressed with PSNR between 18 and 29 dB.

## APPENDIX

### Details of JPEG and JPEG2000 Compression

#### Peak signal-to-noise ratio.

The peak signal-to-noise ratio (PSNR) was used to quantify image fidelity loss in the presence of compression artifacts. PSNR was calculated as described by Equations 1 and 2, where the mean squared error (MSE) was computed between the noise-free original *m* × *n* image *I* and its compressed approximation Î according to:

$$MSE=\frac{1}{mn}\overset{m-1}{\underset{i=0}{\sum }}\overset{n-1}{\underset{j=0}{\sum }}{\left[I\left(i,j\right)-Î\left(i,j\right)\right]}^{2}.$$ (1)

PSNR is then defined as:

$$PSNR=10{\text{log}}_{10}\;\left(peakval^{2}/MSE\right),$$ (2)

where *peakval* is the maximum pixel value possible depending on the data type. In this case, it would be the highest value (pixel intensity) presented on the image.

#### Image compression.

Images were compressed at increasing levels and saved into 2 common lossy formats, JPEG and JPEG2000. JPEG2000 images were encoded from original TIFF images for all use cases via the OpenJPEG library. The quality of the output was determined by specifying the desired PSNR. Additionally, JPEG2000 compression algorithm supports tile-based compression, wherein small blocks of the images are individually compressed and stitched together. We applied various tile sizes for JPEG2000 compression for the experiments pertaining to the first use case: tiles of 64 × 64, 128 × 128, 256 × 256, and 512 × 512 were evaluated via deep learning (DL). The trends in performance of the DL approach were consistent, although minor differences across tile size were observed. The optimal tile size observed was 256 × 256, which yielded the highest F score in nuclei segmentation (< 1% difference compared with other tile sizes). Interestingly, 256 × 256 was also the same tile size used in the original TIFF images before our experiment.

JPEG images were compressed with open source software ImageMagick, with the quality specified by the ImageMagick JPEG compression algorithm ranging from an effective range of 1 to 95. Any quality score > 95 shows little difference compared with 95. JPEG compression does not support lossless compression, which means that even if the quality score is set to 100, the compressed image cannot be reconstructed into the original image, whereas JPEG2000 compression supports lossless encoding.

### Training of Convolutional Neural Networks

All 3 of DL models were trained using a fixed batch size of 64. A typical DL training scheme was then used: mean corrected batches were introduced into the network, an error derivative was calculated, and this was back-propagated through the network by updating the network weights. During training, data set augmentation was enacted by random rotations of {0, 90, 180, 270}, along with random mirroring. All models were trained for 30 epochs using an exponentially annealed learning rate. The final classifier was used for generating the output masks.

Use case 2 (breast lymph node metastasis) saw the application of stain normalization to the input images before training to help address the large heterogeneity in stain presentation unique to that use case. To improve classifier performance, false positive/negative sampling was performed via the hypersampling of these regions from probability masks generated from training data.

### Details of Image Quality at Threshold Compression Level for Acceptable Performance

The goal of this study was to find operating point extrema for both DL approaches and pathologists, not to suggest that those extrema be used in practice. Given the high image degradation at these extremes, in practice one would instead aim to operate at a level where sufficient detail is consistently present. An important takeaway from this study is that given the robust performance of both humans and DL in the context of visually appreciable compression artifacts, there is flexibility in the selection of a practical compression level without fear of a sharp drop in performance. That said, when comparing at high magnification the difference between the original image (PSNR, 90 dB) and an image at an 80% compression ratio (PSNR, 40 dB), only subtle differences could be noted. However, when pushing toward 95% compression ratio (PSNR, 30 dB), as the reviewer suggests, notable artifacts are introduced (Appendix Fig A1). The figure shows that the low-compression image has low-magnitude differences homogenously dispersed through the image, whereas the high-compression image has high-magnitude differences, often localized around regions of higher complexity. From our observation of DL results, as well pathologists’ rationales in picking out the threshold compression levels, these changes are the changes on which both systems rely. Performance drops significantly as a result of ambiguity after hitting these threshold levels. As such, we believe the evidence suggests that higher compression ratios than those currently used may potentially be used without significant modifications to the image.

### Improvement of DL Performance by Using Lossy Compression As Form of Augmentation

An experiment was performed to explore whether using lossy compression as a form of augmentation could improve the performance of the DL models. The nuclei segmentation model in use case 1 was retrained with lossy compression added to the data set augmentation, where every single batch, before being passed to training, was compressed with JPEG2000 with a PSNR setting from 18 to 40 dB (the same levels the previous experiments used). This gave us a training database 24 times as large as the original, which also resulted in a significantly extended training time (approximately 20 times longer). The results are shown in Appendix Figures A2 and A3. Interestingly, a slight improvement (0.0073) in the F score of model performance on uncompressed testing data was observed. Moreover, marginal (< 0.015) improvement in F score was observed for images compressed with PSNR between 30 and 100 dB. Minor to significant improvement (0.005-0.093) in F score was observed on more heavily compressed images using a PSNR setting of 18 to 29 dB. This improvement was observed to be within a normal distribution across the PSNR levels, where most improvement (0.093) was observed at a compression level of PSNR of 24 dB.

We believe the reason behind this observation is because compression artifacts become more apparent and obvious as PSNR decreased until the point at which (PSNR, 30 dB) the textural and gradient attributes of the image (eg, edges, color, lines) are significantly obfuscated. Adding compression noise to the training images slightly increased the generalizability of the model, which in turn resulted in a minor improvement of model performance on uncompressed testing data. However, images compressed with PSNR within the range of 23 to 30 dB suffered from compression artifacts such as ringing, blocking, and color distortion. These artifacts abruptly altered the shape, texture, and edge attributes of the tissue images. However, the nuclear boundaries were still detectable for this range of compression. As a result, compression levels of 22 to 30 dB benefited the most from compression-based augmentation. Finally, images that were compressed with PSNR from 18 to 21 dB underwent a more dramatic image alteration to the point where the individual nuclei were barely visible. Thus, little meaningful information was left to support the DL networks, and little gain in performance was observed from compression augmentation.

Even though there were significant improvements in DL performance on more heavily compressed images, the added value behind using lossy compression as a form of augmentation does not seem to be significant. As discussed previously, compression operations with PSNR below 30 dB are not recommended. Such lossy compression resulted in strong compression artifacts and substantial distortion to original image. Additionally, < 5% compression ratios were achievable by invoking a compression PSNR of 30 to 25 dB.

Additional DL parameters used: number of parameters, 16,777,216; base learning rate, 0.01; solver type, SGD; batch size, 64; training epochs, 30.

### Features Derived From Nuclei Segmentation

Features derived from nuclei segmentation were as follows: area standard deviation, area average, area minimum/maximum, area disorder, perimeter standard deviation, perimeter average, perimeter minimum/maximum, perimeter disorder, chord standard deviation, chord average, chord minimum/maximum, chord disorder, side length minimum/maximum, side length standard deviation, side length average, side length disorder, triangle area minimum/maximum, triangle area standard deviation, triangle area average, triangle area disorder, MST edge length average, MST edge length standard deviation, MST edge length minimum/maximum, MST edge length disorder, area of segmentation, number of nuclei, density of nuclei, average distance to 3 nearest neighbors, average distance to 5 nearest neighbors, average distance to 7 nearest neighbors, average nearest neighbors in a 10-pixel radius, average nearest neighbors in a 20-pixel radius, average nearest neighbors in a 30-pixel radius, average nearest neighbors in a 40-pixel radius, average nearest neighbors in a 50-pixel radius, standard deviation nearest neighbors in a 10-pixel radius, standard deviation nearest neighbors in a 20-pixel radius, standard deviation nearest neighbors in a 30-pixel radius, standard deviation nearest neighbors in a 40-pixel radius, standard deviation nearest neighbors in a 50-pixel radius, disorder of nearest neighbors in a 10-pixel radius, disorder of nearest neighbors in a 20-pixel radius, disorder of nearest neighbors in a 30-pixel radius, disorder of nearest neighbors in a 40-pixel radius, disorder of nearest neighbors in a 50-pixel radius, number of nodes, number of edges, average degree, everage eccentricity, diameter, radius, average eccentricity 90%, diameter 90%, radius 90%, average path length, clustering coefficient C, clustering coefficient D, clustering coefficient E, number of connected components, giant connected component ratio, average connected component size, number of isolated nodes, percentage of isolated nodes, number of end points, percentage of end points, mean edge length, standard deviation of edge length, skewness of edge length, and kurtosis of edge length.
